# Protective Effects of Cannabidiol (CBD) against Qxidative Stress, but Not Excitotoxic-Related Neuronal Cell Damage—An In Vitro Study

**DOI:** 10.3390/biom14050564

**Published:** 2024-05-09

**Authors:** Danuta Jantas, Monika Leśkiewicz, Magdalena Regulska, Magdalena Procner, Piotr Warszyński, Władysław Lasoń

**Affiliations:** 1 Maj Institute of Pharmacology, Polish Academy of Sciences, Department of Experimental Neuroendocrinology, PL 31343 Krakow, Poland; leskiew@if-pan.krakow.pl (M.L.); regulska@if-pan.krakow.pl (M.R.); procner@if-pan.krakow.pl (M.P.); lason@if-pan.krakow.pl (W.L.); 2 Jerzy Haber Institute of Catalysis and Surface Chemistry, Polish Academy of Sciences, PL 30239 Krakow, Poland; piotr.warszynski@ikifp.edu.pl

**Keywords:** cannabidiol, neuroprotection, neurotoxicity, SH-SY5Y cells, primary cortical neurons, hydrogen peroxide, glutamate, 6-hydroxydopamine

## Abstract

Cannabidiol (CBD) appears to possess some neuroprotective properties, but experimental data are still inconsistent. Therefore, this in vitro study aimed to compare the effects of CBD in a wide range of concentrations on oxidative stress and excitotoxic-related cell damage. Results showed that low concentrations of CBD ameliorated the H_2_O_2_-evoked cell damage of primary cortical neuronal cell culture. However, higher concentrations of CBD alone (5–25 μM) decreased the viability of cortical neurons in a concentration-dependent manner and aggravated the toxic effects of hydrogen peroxide (H_2_O_2_). Neuroprotection mediated by CBD in primary neurons against H_2_O_2_ was not associated with a direct influence on ROS production nor inhibition of caspase-3, but we found protective effects of CBD at the level of mitochondrial membrane potential and DNA fragmentation. However, CBD had no protective effect on the glutamate-induced cell damage of cortical neurons, and in higher concentrations, it enhanced the toxic effects of this cell-damaging factor. Likewise, CBD, depending on its concentration, at least did not affect or even enhance cortical cellular damage exposed to oxygen–glucose deprivation (OGD). Finally, we showed that CBD in submicromolar or low micromolar concentrations significantly protected human neuronal-like SH-SY5Y cells against H_2_O_2_- and 6-hydroxydopamine (6-OHDA)-induced cell damage. Our data indicate that CBD has a dual effect on oxidative stress-induced neuronal death-in low concentrations, it is neuroprotective, but in higher ones, it may display neurotoxic activity. On the other hand, in excitotoxic-related models, CBD was ineffective or enhanced cell damage. Our data support the notion that the neuroprotective effects of CBD strongly depend on its concentration and experimental model of neuronal death.

## 1. Introduction

Cannabidiol (CBD) is a psychoactive phytocannabinoid devoid of addictive potency, with potential beneficial effects in the treatment of neurological and neuropsychiatric disorders [1,2,3,4,5]. CBD is a multitargeted drug with neuroprotective and anti-inflammatory properties, which shows affinity to various molecular targets, comprising peroxisome proliferator-activated receptor gamma (PPARγ), transient receptor potential vanilloid receptors (TRPV), G protein-coupled receptor 55 (GPR55), 5-HT_1A_ serotonin receptors, enzymes, transporters and ion channels, and negligible affinity to cannabinoid CB1 and CB2 receptors [6,7,8,9,10]. Furthermore, CBD was reported to interfere with pivotal mechanisms of neuronal death in neurodegenerative disorders such as oxidative stress and excitotoxicity [11,12,13]. Moreover, CBD is used for the treatment of drug-resistant epilepsies [10]. Oxidative stress results from an imbalance between the overproduction of reactive oxygen/nitrogen species (ROS/RNS) and a deficit in endogenous antioxidative systems. Excessive production of ROS/RNS evokes peroxidation of the polyunsaturated fatty acids, nitration and carbonylation of proteins, and oxidation of DNA, leading to cellular damage [14]. Excitotoxicity is characterized by excessive glutamate release and overstimulation of ionotropic glutamate receptors like NMDA (N-methyl-D-aspartate), AMPA (α-amino-3-hydroxy-5-methyl-4-isoxazolepropionic acid), and KA (kainic acid) receptors, resulting in a profound increase in intracellular calcium ion levels, which ultimately leads to cell death [15].

Regarding CBD interference with oxidative stress, it was shown that this compound protected rat cortical neuronal cell culture against tertbutyl hydroperoxide toxicity in a concentration-dependent way with similar efficiency as an antioxidant butylated hydroxytoluene (BHT, 2,6-bis(1,1-dimethyl ethyl)-4-methylphenol) [11]. Moreover, in the model of glutamate-induced toxicity, it was more protective than other strong antioxidants such as ascorbate or β-tocopherol [11]. Recently, it was reported that CBD ameliorated oxidative stress-related damage induced using hydrogen peroxide (H_2_O_2_) and mitochondrial toxin-rotenon in primary cerebellar granule neurons [16]. Of note, these investigators found that higher concentrations of CBD and some other cannabinoids decreased the viability of neural cells [16]. These findings suggest that attenuation or aggravation of oxidative stress-related cell damage by CBD is strongly concentration-dependent.

Regarding excitotoxicity, it was observed that CBD in concentrations up to 10 μM protected cortical neurons in vitro against glutamate toxicity regardless of whether the insult was mediated by NMDA, AMPA, or kainate receptors [11]. Other authors reported that CBD prevented NMDA-induced retinal neurotoxicity and that the mechanism of this action involved peroxynitrite [17]. CBD also reduced ROS accumulation and lipid peroxidation in the diabetic retinopathy model [18] and in the in vitro model of Alzheimer’s disease-PC12 cells exposed to β-amyloid [19]. However, data on the effects of CBD on oxidative stress and excitotoxic processes are often inconsistent. Moreover, CBD was reported to exert neuroprotective or neurotoxic effects depending on experimental settings and the neuronal model [13]. Therefore, in our study, we compared the neuroprotective efficacy of CBD in a wide range of concentrations for in vitro models of neural cell damage induced using prooxidative (hydrogen peroxide—H_2_O_2_, 6-hydroxydopamine—6-OHDA) and excitotoxic (glutamate—Glu, oxygen-glucose deprivation—OGD) insults. For this purpose, we employed mouse primary neuronal cell cultures and a human neuronal-like model, neuroblastoma SH-SY5Y cells. Primary neuronal cell cultures obtained from rodent brains are widely used as a screening platform for neurotoxicity and neuroprotection [20]. There are several in vitro reports showing its utility for testing the neuroprotective potency of CBD against various cell-damaging factors, including prooxidative or excitotoxic insults [13]. SH-SY5Y cells are frequently used in human cellular models to study mechanisms and therapeutic strategies for neurodegenerative diseases, especially Parkinson’s disease [21]. Due to their dopaminergic phenotype, they are susceptible to dopaminergic neurotoxins (e.g., 6-OHDA). They were reported to express an endocannabinoid system [22] and have been used as a model for testing the neuroprotective potency of CBD [23,24].

## 2. Materials and Methods

### 2.1. Chemicals

Neurobasal A (w/o phenol red), supplement B27 (w/o antioxidants), high glucose DMEM (Dulbecco’s Modified Eagle’s Medium), FluoroBrite™ DMEM, heat-inactivated FBS (fetal bovine serum), 0.25% trypsin/EDTA and penicillin/streptomycin solution were obtained from Gibco (Life Technologies Ltd., Paisley, UK). The Cytotoxicity Detection Kit and Cell Proliferation Reagent WST-1 were purchased from Roche Diagnostics GmbH (Mannheim, Germany). Caspase-3 (Ac-DEVD-AMC) fluorogenic substrate was from Enzo Life Sciences (New York, NY, USA). CM-H_2_DCFDA, fluorescently labeled secondary antibodies (goat anti-mouse IgG (H+L) AlexaFluor^®^ 488 and donkey anti-rabbit IgG (H+L) AlexaFluor^®^ 568)) and ProLong^®^Gold antifade reagent were from Molecular Probes (Life Technologies Corporation, Eugene, OR, USA). Cannabidiol (CBD, 2-[1R-3-methyl-6R-(1-methylethenyl)-2-cyclohexen-1-yl]-5-pentyl-1,3-benzenediol) was purchased from Cayman Chemical (Ann Arbor, Michigan, USA). All other reagents were from Sigma (Sigma-Aldrich Chemie GmbH, Taufkirchen, Germany).

### 2.2. Primary Neuronal Cell Cultures

Pregnant CD1 mice were obtained from Charles River Laboratories (Sulzfeld, Germany). Primary neuronal cell cultures were prepared from 15/16 days embryos using the previously described method [25]. The protocol for generating the primary neuronal cell cultures is in line with the European Union (Directive 2010/63/EU, amended by Regulation (EU) 2019.1010) guidelines on the ethical use of animals. All experiments were conducted according to the principles of the Three Rs, and all efforts were made to minimize the number of animals used and their suffering. The cortical tissue was trypsinized (0.1% trypsin in PBS w/o Ca^2+^/Mg^2+^) at room temperature for 20 min, and cells were counted in the Bürker chamber. Next, they were seeded at densities 6 × 10^4^ and 3 × 10^5^ cells per well in poly-L-ornithine (0.05 mg/mL)-covered 96- and 24-well plates, respectively. For the first two days, the cell culture medium (Neurobasal A medium, 2 mM L-glutamine, 0.4% B27, 0.06 μg/mL penicillin, and 0.1 μg/mL streptomycin) was supplemented with 5% FBS. The cells were cultured at 37 °C in a humidified atmosphere containing 5% CO_2_ for eight days prior to experimentation with medium exchange every two days. This procedure typically yields cultures that contain about 80% of neurons and about 20% of astrocytes, as was measured using MAP-2 and GFAP immunofluorescence.

### 2.3. SH-SY5Y Cell Culture

Human neuroblastoma cell line SH-SY5Y (ATCC, CRL-2266, Manassas, VA, USA) was cultured in DMEM containing 10% of heat–inactivated FBS and 1% of 100 U/mL penicillin, and 0.1 mg/mL streptomycin in 75-cm^2^ culture flasks and were grown in a humidified chamber with 5% CO_2_ at 37 °C. When the cells reached about 80% confluency, they were seeded into 96-well plates at a density of 4 × 10^4^ cells per well. Twenty-four hours before cell treatment, the culture medium was changed to a serum-free medium containing a 1% N_2_ supplement. For experiments, cells between passage numbers 3–20 were used.

### 2.4. Cell Treatment

The primary neuronal cell cultures growing on 96-well plates were first treated with CBD alone at concentrations 1–25 μM to assess the biosafety of the tested compounds. Next, all these cells were pretreated with CBD (0.01–25 μM) for 30 min, followed by H_2_O_2_ (0.2 mM) or glutamate (Glu, 1 mM) exposure for the next 24 h. We chose optimal concentrations of particular cell-damaging agents (H_2_O_2_ and Glu) and time of exposure (24 h) based on our previous study [25]. Moreover, we examined the effect of CBD (0.01–0.5 μM) in primary neurons against cell damage evoked by oxygen-glucose deprivation (OGD) as was optimized and described in our previous study [26]. CBD was given under three schedules-before OGD, before and after OGD, and after OGD. Briefly, the cells were washed twice and placed in glucose-free Earle’s balanced salt solution (EBBS, pH 7.4, purged with a 95% N_2_ and 5% CO_2_ gas mixture for 5 min), treated with CBD (for experimental groups: before OGD; before and after OGD) and placed in an airtight chamber (Billups-Rothenberg Inc., San Diego, CA, USA) equipped with inlet and outlet valves, and flushed by 95% N_2_ and 5% CO_2_ gas mixture for 5 min. The chamber was sealed and placed into a humidified incubator at 37 °C for 180 min with additional chamber flushing with 95% N_2_ and 5% CO_2_ gas mixture after 90 min. During such a procedure, the oxygen concentration in the chamber is maintained at 0.1%. Control cells were incubated in EBBS containing 5 mM glucose in a normoxic incubator for the same time and with a similar treatment with CBD. OGD was terminated by removing the cultures from the airtight chamber, exchanging EBBS from OGD-exposed and normoxia cells with the pre-warmed Neurobasal culture medium, treatment of cells with CBD (experimental groups: before and after OGD; after OGD) and cultured for next 24 h under the normoxic conditions (reoxygenation period). We employed the NMDA receptor antagonist, MK-801 (1 μM), as a positive control for Glu and OGD models. In the OGD model, MK-801 was given two times, before and after OGD.

The SH-SY5Y cells were treated with CBD alone (0.01–5 μM) for 24 h or pretreated for 30 min with CBD (0.01–5 µM) followed by 24 h exposure to H_2_O_2_ (0.3 mM) or 6-OHDA (0.1 mM). The optimal concentrations of cell-damaging factors and time of treatment were chosen on the basis of our previous work [25].

Stock solution of CBD (10 mM) was prepared in 100% ethanol and kept at −80 °C. The final solutions of this compound were prepared in 70% ethanol and kept at −20 °C. Ac-DEVD-CHO (20 mM) stock solution was prepared in DMSO, and its final solution was prepared in distilled water. The H_2_O_2_ (100 mM) stock solution was prepared freshly from stabilized 30% hydrogen peroxide diluted to the final concentration in distilled water. The 6-OHDA (10 mM) stock solution was prepared in distilled water on the day of cell treatment. The Glu (100 mM) stock solution was prepared in 100 mM NaOH immediately before use. The buffers for the OGD model were prepared according to the procedure described in our previous study [26]. Each experimental set of the control cultures was supplemented with the appropriate vehicle (70% ethanol), and the solvent was present in cultures at a final concentration of 0.1%. All agents were added to the culture medium under light-limited conditions to avoid potential light-induced cytotoxicity.

### 2.5. MTT Reduction Cell Viability Assay

The assessment of cell viability of primary neuronal cell cultures growing in 96-well format was performed by 3-[4,5-dimethylthiazol-2-yl]-2,5-diphenyltetrazolium bromide (MTT) assay according to the procedure described previously [25]. The data were normalized to vehicle-treated cells (100%) and are presented as a mean ± SEM from 3–8 independent experiments with 3–5 replicates each.

### 2.6. WST-1 Cell Viability Assay

Cell viability in SH-SY5Y cells viability was estimated using Water-Soluble Tetrazolium 1 (WST-1) cell proliferation reagent. The stable tetrazolium salt WST-1 was cleaved into formazan dye according to mitochondrial dehydrogenase activity. The amount of formazan dye formed directly correlates to the number of metabolically active cells. Twenty-four hours after cell treatment, 5 μL of WST-1 solution was added to the cell culture for 30–60 min. Finally, absorbance was read at 450 nm with a reference wavelength level of 650 nm using an Infinite M200PRO microplate reader (Tecan Austria GmbH, Grodig, Austria). The intensity of the red color formed in the assay is proportional to the number of viable cells. The data were normalized to vehicle-treated cells (100%) and are expressed as the mean ± SEM from 2–3 independent experiments with 3–5 replicates.

### 2.7. LDH Release Assay

The lactate dehydrogenase (LDH) released into culture media was used to assess the cytotoxic potential of the tested compounds with the Cytotoxicity detection kit (Roche) as described previously [25]. The medium was collected from the plates used for cell viability assessment. The data were normalized to vehicle-treated cells (100%) and are presented as a mean ± SEM from 2–8 independent experiments with 3–5 replicates each.

### 2.8. Measurement of Intracellular Reactive Oxygen Species (ROS)

The intracellular ROS level was measured with 5-(and 6-)-chloromethyl-2′,7′-dichlorodihydrofluorescein diacetate, acetyl ester (CM-H_2_DCFDA) as described previously [27]. The cells were washed with pre-warmed FluoroBrite™ DMEM and loaded with 5 μM CM-H_2_DCFDA dissolved in FluoroBrite™ DMEM and located for 10 min in an incubator. Next, the cells were treated with CBD (0.5–5 μM), NAC (1 mM), and H_2_O_2_ (1 mM) for 50 min. Afterward, the cells were washed twice with pre-warmed FluoroBrite™ DMEM, and the fluorescence was measured in a microplate multi-well reader (Infinite^®^ M200 PRO, Tecan Austria GmbH, Grodig, Austria) with excitation and emission wavelengths of 485 nm and 535 nm, respectively. The data were normalized to the vehicle-treated cells (100%) and are presented as the mean ± SEM from 2 independent experiments, two plates in each experiment with 3–5 replicates.

### 2.9. Measurement of Mitochondrial Membrane Potential (MMP)

The tetramethylrhodamine ethyl ester (TMRE) was used for MMP measurement, as described previously [27]. The cells were pretreated for 30 min with CBD (0.01–5 μM) followed by 6 h exposure to H_2_O_2_ (0.2 mM). Next, the cells were washed with pre-warmed FluoroBrite™ DMEM loaded with 200 nM TMRE dissolved in FluoroBrite™ DMEM and located in an incubator for 20 min. After two washings with pre-warmed FluoroBrite™ DMEM, the fluorescence was measured in a multi-well microplate reader (Infinite^®^ M200 PRO, Tecan Austria GmbH, Grodig, Austria) with excitation and emission wavelengths of 540 nm and 595 nm, respectively. Data were normalized to vehicle-treated cells (100%) and are presented as the mean ± SEM from 2 independent experiments, two plates in each experiment with 3–5 replicates.

### 2.10. Caspase-3 Activity Assay

Primary neuronal cell cultures growing in 96-well format were treated with CBD (0.01–5 μM) and H_2_O_2_ (0.2 mM) for 9 h. Caspase-3 inhibitor, Ac-DEVD-CHO (20 μM), was used as a positive control for the measurement. Caspase-3 activity was measured in cell lysates using the fluorogenic substrate Ac-DEVD-AMC (50 μM) as described previously [27]. The data were normalized to vehicle-treated cells (100%) and presented as the mean ± SEM from 4 independent experiments with 3–5 replicates each.

### 2.11. Immunofluorescence and Hoechst 33342 Staining

Primary neuronal cell cultures growing on poly-L-ornithine (0.05 mg/mL)-covered round cover glasses (diameter 11 mm) in 24-well plate format after 30 min pretreatment with CBD (0.1–1 μM) followed by 24 h exposure to H_2_O_2_ (0.2 mM) were fixed with 4% paraformaldehyde and immunostained with neuronal (mouse anti-MAP-2 antibody) and astrocyte (rabbit anti-GFAP antibody) markers as described previously [27]. After staining with fluorescently labeled secondary antibodies (goat anti-mouse IgG (H+L) AlexaFluor^®^ 488 and donkey anti-rabbit IgG (H+L) AlexaFluor^®^568), the cells were counterstained with nuclear dye Hoechst 33342 and mounted in ProLong^®^Gold antifade reagent). The samples were imaged with the inverted fluorescence microscope (AxioObserver, Carl Zeiss, Jena, Germany) equipped with a black-white camera (Axio-CamMRm, Carl Zeiss, Jena, Germany) with the excitation wavelengths 470 nm, 555 nm, and 365 nm for AlexaFluor^®^488, AlexaFluor^®^568 and Hoechst 33342, respectively. Five microphotographs were taken for all experimental groups under each fluorescence panel, and images were collected from 4 independent experiments. The number of pyknotic nuclei (fragmented or/and condensed) and healthy (uniformly stained) nuclei were counted semi-automatically from Hoechst 33342 images using AxioVision Rel. 4.8.2 SP2 (06-2012) Software (Carl Zeiss, Jena, Germany). Data were calculated as a percentage of cells with pyknotic nuclei relative to the whole cell number and presented as the mean ± SEM. A similar method was used to estimate the percentage of MAP-2 and GFAP-positive cells in all experimental groups. Representative images of cells stained with neuronal and glial markers and counterstained with Hoechst 33342 dye were acquired using a Leica TCS SP8 confocal microscope (Leica Microsystems GmbH, Wetzlar, Germany) equipped with a white light laser (WLL), using LAS X software (version 3.5.7.23225). Neuronal and glial fluorescence images were recorded using spectral detectors (HyD), and cell nuclei were recorded using a photomultiplier tube (PMT). Images were captured using an HC PL APO CS2 20x/0.75 DRY lens at zoom 2. Digital image dimensions: 2048 × 2048 pixels, pixel size 142 nm. A bidirectional scan was performed with triple line averaging for better quality. Each channel was scanned separately using the sequential mode: Hoechst 33342 dye excitation using 405 nm laser, emission recorded at range 410 nm–504 nm; AlexaFluor^®^488 dye excitation using 499 nm laser, emission recorded at range 504 nm–548 nm; AlexaFluor^®^568 dye excitation using 553 nm laser, emission recorded at range 558 nm–725 nm.

### 2.12. Statistical Analysis

Data were analyzed using the one-way analysis of variance (one-way ANOVA) and post hoc Duncan test for multiple comparisons using the Statistica 13 software (StatSoft Inc., Tulsa, OK, USA). The statistical significance was assumed with *p* < 0.05.

## 3. Results

### 3.1. The Neuroprotective Effects of CBD in Primary Neuronal Cell Cultures

**Figure 1:**
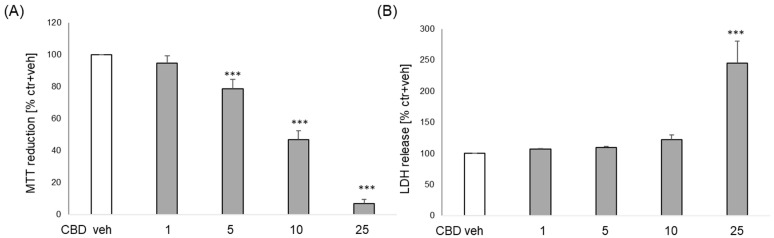
Biosafety assessment of cannabidiol CBD) in primary neurons. The cells were treated for 24 h with CBD (1–25 μM). Cell viability (**A**) and cytotoxicity (**B**) were estimated by MTT reduction and LDH release assays, respectively. The data were normalized to vehicle-treated cells and are presented as the mean ± SEM from 3–8 independent experiments with 3–5 replicates. *** *p* < 0.001 vs. vehicle-treated cells.

In primary neuronal cell cultures, CBD at concentrations 5, 10, and 25 μM evoked a significant and concentration-dependent decrease in cell viability (from 20–95%) (Figure 1A), and at the concentration of 25 μM increased by about 2.5-fold LDH release when compared to vehicle-treated cells (Figure 1B).

**Figure 2:**
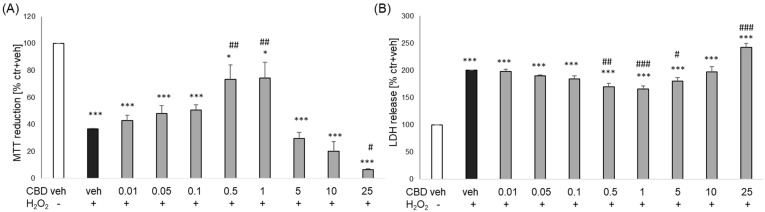
Neuroprotective effects of cannabidiol (CBD) against cell damage evoked by H_2_O_2_ in primary neurons. The cells were treated for 24 h with CBD (0.01–25 μM) and H_2_O_2_ (0.2 mM). Cell viability (**A**) and cytotoxicity (**B**) were estimated by MTT reduction and LDH release assays, respectively. The data were normalized to vehicle-treated cells and are presented as the mean ± SEM from 3–7 independent experiments with 3–5 replicates. * *p* < 0.05 and *** *p* < 0.001 vs. vehicle-treated cells; # *p*< 0.05, ## *p* < 0.01 and ### *p* < 0.001 vs. H_2_O_2_-treated cells.

In the cell damage model induced using H_2_O_2_ in primary neurons, we observed over 60% decrease in cell viability, which was reduced by half by CBD at concentrations 0.5 and 1 μM (Figure 2A). We also observed the neuroprotective effect of CBD at the level of the cytotoxic marker, where CBD at concentrations 0.5–5 μM partially (by about 10–18% of H_2_O_2_-induced changes) decreased the H_2_O_2_-induced LDH release (Figure 2B). We detected an enhanced cell-damaging effect of H_2_O_2_ by CBD at a concentration of 25 μM, confirmed using MTT reduction and LDH release assays (Figure 2).

**Figure 3:**
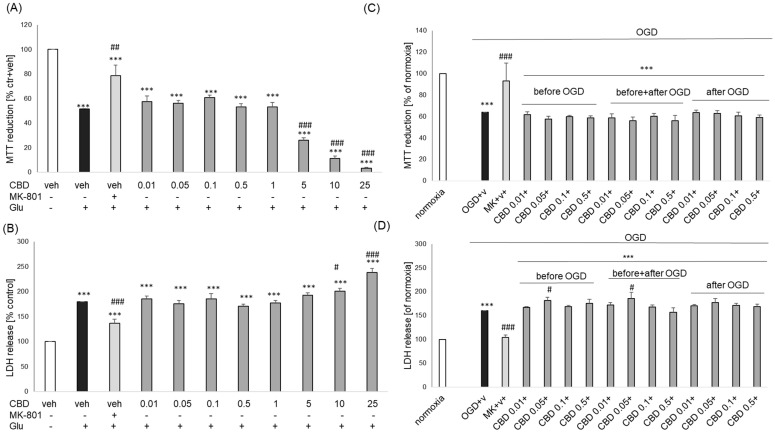
The effects of cannabidiol (CBD) on the glutamate (Glu, (**A**,**B**))- or oxygen-glucose deprivation (OGD, (**C**,**D**))-induced cell damage in primary neurons. (**A**,**B**) The cells were treated with CBD (0.01–25 μM) in combination with Glu (1 mM) for 24 h. (**C**,**D**) The cells were treated with CBD (0.01–0.5 μM) under three schedules (before OGD, before + after OGD, after OGD) combined with a 3 h OGD procedure and 24 h of reoxygenation period. NMDA receptor antagonist MK-801 (1 μM) was used as a positive control for both cell damage models. Cell viability (**A**,**C**) and cytotoxicity (**B**,**D**) were measured by MTT reduction and LDH release assays, respectively. The data were normalized to vehicle-treated cells and presented as the mean ± SEM from 3–7 independent experiments with 3–5 replicates. *** *p* < 0.001 vs. vehicle-treated cells; # *p*< 0.05, ## *p* < 0.01 and ### *p* < 0.001 vs. Glu/OGD-treated cells.

In the cell damage model induced using Glu in primary neurons, we observed about a 50% decrease in cell viability and around a 1.8-fold increase in released LDH level when compared to vehicle-treated cells, which was significantly attenuated by NMDA receptor antagonists, MK-801 (1 μM) (Figure 3A,B). None of the tested CBD concentrations (0.01–25 μM) were protective against Glu-evoked cell damage (Figure 3A,B). However, this compound in concentrations over 5 μM significantly increased the Glu-induced decrease in cell viability (Figure 3A), and for concentrations above 10 μM, it also enhanced the Glu-stimulated LDH release (Figure 3B).

The OGD model is commonly used for in vitro experimental modeling of stroke where excitotoxic processes are predominant [25,26,27]. In such a model in primary neuronal cell culture, we observed neuroprotective effects of NMDA receptor antagonist MK-801 (1 μM, given before + after OGD) at the level of cell viability (Figure 3C) as well as the LDH release (Figure 3D). We did not find any protection mediated by CBD at all tested concentrations (0.01–0.5 μM) under any of the tested administration schedules (before OGD, before + after OGD, after OGD), as was confirmed in both biochemical assays (Figure 3C,D). In the LDH release assay, we observed a slight (by about 15% of OGD-induced changes) increase in the cytotoxic effect of OGD by CBD at a concentration of 0.05 μM when it was given before OGD and before + after OGD (Figure 3D).

### 3.2. Verification of Potential Mechanisms Involved in the CBD-Mediated Neuroprotection

**Figure 4:**
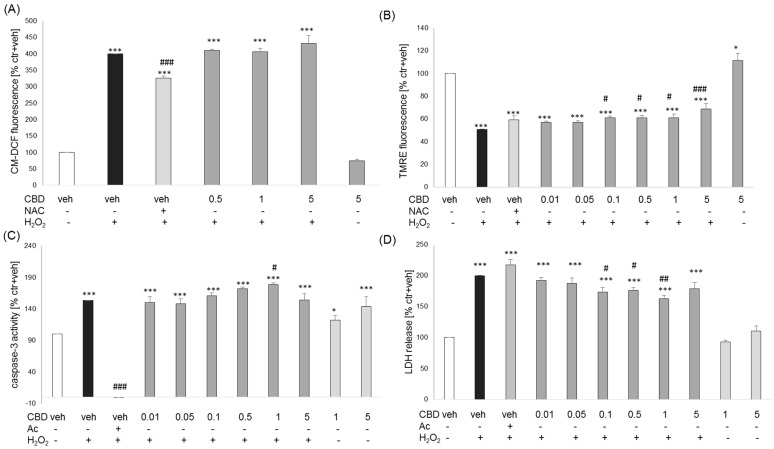
The effects of cannabidiol (CBD) on the H_2_O_2_-evoked changes in reactive oxygen species level (ROS, (**A**)), mitochondrial membrane potential (MMP, (**B**)), caspase-3 activity (**C**), and cytotoxicity (**D**) in primary neuronal cell cultures. (**A**) ROS production was assayed with a CM-H_2_DFFDA probe, as described in details found in the Material and Methods section. (**B**) MMP measurement was performed after 6 h of treatment of cells with CBD (0.01–5 μM), NAC (1 mM), and H_2_O_2_ (0.2 mM) employing a TMRE fluorescence probe. (**C**,**D**) Caspase-3 activity and cytotoxicity measurements were performed in cells treated for 9 h with CBD (0.01–5 μM) and H_2_O_2_ (0.2 mM). A caspase-3 inhibitor, Ac-DEVD-CHO (Ac, 20 μM), was used as a positive control to the assay. After treatment, in cell lysates, caspase-3 activity (**C**) was measured using the fluorogenic substrate Ac-DEVD-AMC, and cytotoxicity was assessed in the cell culture medium (LDH test, (**D**)). Data after normalization to vehicle-treated cells (100%) are presented as a mean ± SEM. * *p* < 0.05 and *** *p* < 0.001 vs. vehicle-treated cells; # *p* < 0.05, ## *p* < 0.01 and ### *p* < 0.001 vs. H_2_O_2_- treated cells.

Since previous data indicated the direct antioxidant potency of CBD [11], first, we verified if CBD has any direct effect on ROS production evoked by H_2_O_2_ in primary neuronal cell cultures, which was assessed by CM-DCF fluorescence. We found that H_2_O_2_ (1 mM) increased about 4-fold the CM-DCF fluorescence when compared to vehicle-treated cells, which was significantly reduced by antioxidant NAC (1 mM) but not by any of the tested concentrations of CBD (0.5–5 μM) (Figure 4A). CBD alone (5 μM) did not influence the basal intracellular ROS level compared to vehicle-treated cells (Figure 4A).

The H_2_O_2_-evoked cell damage in primary neuronal cell cultures is associated with MMP decrease, as evidenced in our previous studies [25,27]. We found that CBD alone at concentration 5 μM significantly increased TMRE fluorescence (by about 11%) when compared to vehicle-treated cells and at concentrations 0.1–5 μM slightly (about 20–37% of H_2_O_2_ effects) attenuated the H_2_O_2_-evoked decline in MMP (Figure 4B).

Mitochondrial collapse induced by H_2_O_2_ could activate apoptotic processes in neuronal cells [25,27,28]. Thus, in the next step, we tested the possible effects of CBD on the apoptotic marker caspase-3. We found about 1.5-fold increase in caspase-3 activity after 9 h of treatment with H_2_O_2_ (0.2 mM), which was completely inhibited by caspase-3 inhibitor, Ac-DEVD-CHO (20 μM) but not attenuated by any tested concentrations of CBD (0.01–5 μM) (Figure 4C). Moreover, we observed that CBD alone at concentrations 1 and 5 μM significantly increased basal caspase-3 activity (by about 20 and 40%, respectively), and 1 μM CBD exaggerated the H_2_O_2_-induced enzyme activity (Figure 4C). However, these changes were not reflected in the cytotoxicity level since in LDH release assay after 9 h of treatment, we did not observe any detrimental effects of CBD alone at concentrations 1 and 5 μM, and CBD at concentrations 0.1–1 μM significantly attenuated the H_2_O_2_-evoked LDH release (Figure 4D).

**Figure 5:**
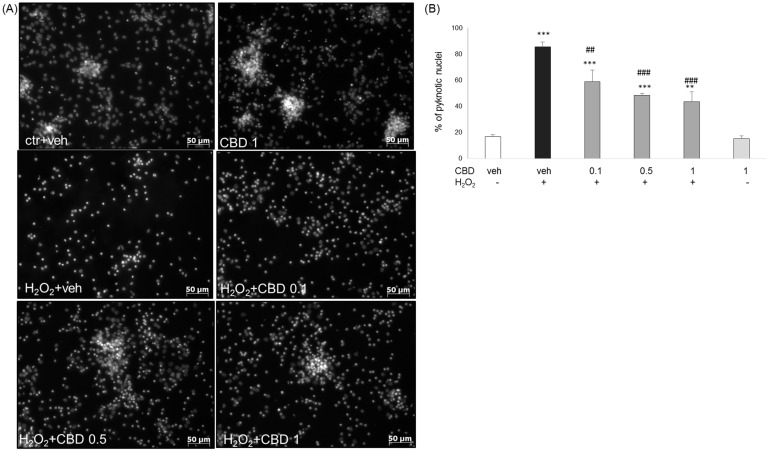
The effect of cannabidiol (CBD) on DNA fragmentation was measured by Hoechst 33342 staining. (**A**) Representative fluorescence images of primary neurons treated with cannabidiol (CBD, 0.1–1 μM) and H_2_O_2_ (0.2 mM) for 24 h and stained with Hoechst 33342 dye. Images were taken with an inverted microscopy AxioObserver. (**B**) Quantitative analysis of DNA fragmentation by counting the cells with pyknotic (fragmented and/or condensed) nuclei. The data are presented as a percentage of pyknotic nuclei ± SEM. ** *p* < 0.01 and *** *p* < 0.001 vs. vehicle-treated cells; ## *p* < 0.01 and ### *p* < 0.001 vs. H_2_O_2_-treated cells.

We also verified the neuroprotective effects of CBD at the level of DNA fragmentation measured by Hoechst 33342 staining. We demonstrated a huge increase in the number of pyknotic (apoptotic and necrotic) nuclei after 24 h treatment with H_2_O_2_ (0.2 mM), which was significantly attenuated by CBD at concentrations 0.1–1 μM (Figure 5).

**Figure 6:**
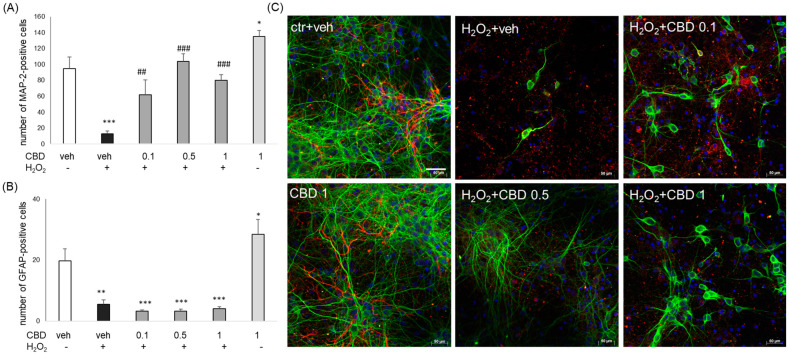
The effect of cannabidiol (CBD) on neuronal (**A**) and glial (**B**) cells. Primary neurons were treated with cannabidiol (CBD, 0.1–1 μM) and H_2_O_2_ (0.2 mM) for 24 h and immunostained with neuronal (MAP-2) and astrocyte (GFAP) marker. Quantitative analysis of MAP-2 and GFAP-positive cells was performed on fluorescence images taken with an inverted microscopy AxioObserver. The data are presented as a number of MAP-2 (**A**) and GFAP (**B**) positive cells per image ± SEM. * *p* < 0.05, ** *p* < 0.01 and *** *p* < 0.001 vs. vehicle-treated cells; ^##^ *p* < 0.01 and ^###^ *p* < 0.001 vs. H_2_O_2_-treated cells. (**C**) Representative confocal images of primary neurons treated with cannabidiol (CBD, 0.1–1 μM) and H_2_O_2_ (0.2 mM) for 24 h. The cells were immunostained with a neuronal marker (MAP-2, green), astrocyte marker (GFAP, red), and nuclear dye Hoechst 33342 (blue) and imaged with a Leica TCS SP8 confocal microscope (Leica Microsystems GmbH, Wetzlar, Germany) equipped with a white light laser (WLL), using LAS X software (version 3.5.7.23225).

Finally, we evidenced neuroprotection by CBD at a morphological level using the MAP-2/GFAP immunofluorescence method. Quantitative analysis of MAP-2- and GFAP-positive cells was performed on fluorescence images taken with an inverted microscopy AxioObserver. The data showed that 24 h of treatment with H_2_O_2_ (0.2 mM) evoked almost complete cell damage of neurons, which was in a concentration-dependent manner attenuated by CBD (0.1–1 μM) (Figure 6A). However, we did not observe any protection by CBD at the level of GFAP-positive cells, which were significantly destroyed by H_2_O_2_ and not protected by CBD (0.1–1 μM) (Figure 6B). Moreover, CBD (1 μM) given alone for 24 h significantly increased the number of MAP-2 and GFAP-positive cells (Figure 6A,B). Representative confocal images are shown in Figure 6C.

### 3.3. The Neuroprotective Effects of CBD in SH-SY5Y Cells

**Figure 7:**
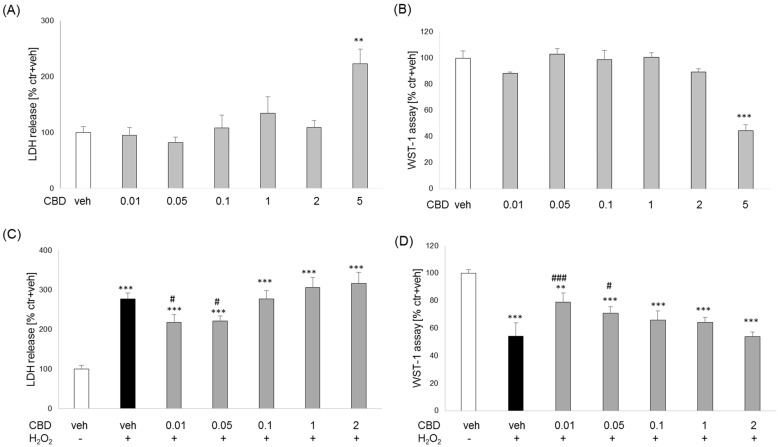
Effects of cannabidiol (CBD) alone and on the H_2_O_2_-induced damage in SH-SY5Y cells. The cells were incubated with CBD (0.01–5 µM) alone for 24 h (**A**,**B**) or were pretreated for 30 min with CBD 0.01–5 μM followed by 24 h exposure to H_2_O_2_ (0.3 mM) (**C**,**D**). The cytotoxicity was estimated by LDH release assay (**A**,**C**) and cell viability by WST-1 assay (**B**,**D**). Data after normalization to vehicle-treated cells (100%) are presented as the mean ± SEM from 2–3 independent experiments with 3–5 replicates. ** *p* < 0.01; *** *p* < 0.001 vs. vehicle-treated cells; # *p* < 0.05 and ### *p* < 0.001 vs. H_2_O_2_-treated cells.

**Figure 8:**
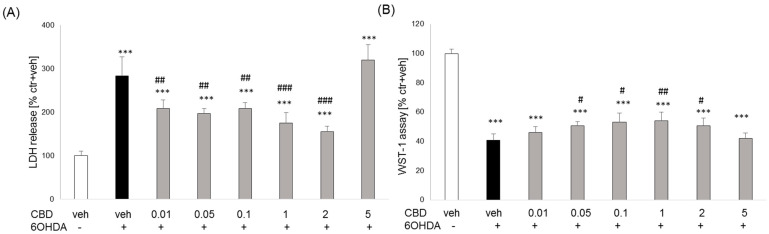
Effect of cannabidiol (CBD) on the 6-OHDA-induced cell damage in SH-SY5Y cells. The cells were pretreated for 30 min with CBD (0.01–5 µM) followed by 24 h exposure to 6-OHDA (0.1 mM). The cytotoxicity was estimated by LDH release assay (**A**) and cell viability by WST-1 assay (**B**). Data after normalization to vehicle-treated cells (100%) are presented as the mean ± SEM from 2–3 independent experiments with 3–5 replicates. *** *p* < 0.001 vs. vehicle-treated cells; # *p* < 0.05, ## *p* < 0.01 and ### *p* < 0.001 vs. 6-OHDA-treated cells.

CBD in the concentration range of 0.01 to 2 μM did not change the level of lactate dehydrogenase in SH-SY5Y cells and did not affect cell viability, while at the higher concentration (5 μM), it significantly increased the release of the enzyme and reduced cell viability (Figure 7A,B). Two toxic agents, H_2_O_2_ and 6-OHDA, were used to assess the neuroprotective effect of CBD. Twenty-four hour incubation of SH-SY5Y cells with H_2_O_2_ and 6-OHDA significantly increased LDH levels (177% and 183% of control, respectively) (Figure 7C and Figure 8A) and reduced cell viability (47% and 48% of control, respectively) (Figure 7D and Figure 8B), as compared to vehicle-treated cells. The neuroprotective effect of CBD on SH-SY5Y cell damage induced by both H_2_O_2_ and 6-OHDA was observed. At the lowest concentrations used (0.01 and 0.05 μM), CBD effectively inhibited the H_2_O_2_-induced cell damage (Figure 7C,D). A stronger inhibitory effect of CBD on the 6-OHDA-stimulated LDH release was observed over a broader range of CBD concentrations (0.01–2 μM) (Figure 8A), while at concentrations 0.05–2 μM, CBD significantly counteracted the effect of 6-OHDA on cell viability (Figure 8B).

## 4. Discussion

Despite the beneficial influence of CBD on some neurological disorders, such as epilepsy and chronic pain, some clinical data suggest that adverse effects of this compound include neurotoxicity [29]. Our study showed that CBD in nanomolar or low micromolar concentrations attenuated neuronal cell damage related to oxidative stress but not excitotoxicity. CBD in higher concentrations (>5 μM), when given alone, significantly decreased the viability of both types of neuronal cells (primary neurons and SH-SY5Y cells), and in primary neurons, also amplified cell damage evoked by prooxidative or excitotoxic insults. Additionally, some clinical data suggest that adverse effects of this compound may include neurotoxicity [29]. Previous studies demonstrated the cell-damaging effects of CBD at concentrations above 5 μM as evidenced in primary rat hippocampal neurons [30], rat cerebral granule cells [16], rat neural progenitor cells [31], or in undifferentiated and neuronally differentiated (with retinoic acid) human SH-SY5Y cells [24,32]. In our study, we demonstrated that in undifferentiated SH-SY5Y cells, CBD at concentration 5 μM is cytotoxic. However, this is in contrast to the findings of some research groups [23,24,32], who showed that up to 10 μM CBD is safe for undifferentiated SH-SY5Y cells. These discrepancies could be explained by the composition of the cell culture medium. In the former studies [23,24,32], a culture medium with 10% FBS content was used, which probably made cells more resistant to the cell-damaging effect of CBD in comparison to the medium containing 1% N_2_ supplement in our study. In some cases, the cytotoxic effect of CBD is associated with the induction of oxidative stress. It could be responsible for the anti-inflammatory action of CBD (>10 μM) in the experimental model of refractory epilepsy, as was evidenced by polymorphonuclear neutrophils (PMNs), which enter the brain parenchyma in the first wave of extravasation after epileptic seizures [33].

Of note, we confirmed the results of a previous study performed by Kim et al. [30] on 1 DIV primary hippocampal neurons that lower concentrations of CBD significantly protected the neurons from the H_2_O_2_-induced death, while higher micromolar concentrations of CBD alone (>5 μM) significantly and in a concentration-dependent manner decreased viability of neuronal cells. As mentioned by Kim et al. [30], the mechanisms of cell death evoked by CBD or H_2_O_2_ are different, and CBD only partly rescued the H_2_O_2_-induced neurite degeneration. In our study, the protective effects of CBD in low concentrations on the H_2_O_2_-induced primary cortical neuron cell damage revealed by biochemical (LDH and MTT assays) and morphological observations (MAP-2 immunostaining) were supported by an increase in MMP. However, we did not observe the protective effects of CBD (0.1–1 μM) on glial cells damaged by H_2_O_2_, as evidenced by the number of GFAP-positive cells. Interestingly, we showed that CBD alone (1 μM) increased the number of MAP-2- and GFAP-positive cells, suggesting the protective effects of this compound under basal conditions in neuronal and glial cells. A decrease in MMP is associated with cytochrome c release during apoptosis and might be an indirect marker for cytochrome c release in cells [34]. Although we did not perform a specific mechanistic study in this respect, the counteracting effect of CBD on the H_2_O_2_-induced loss of MMP suggests an anti-apoptotic effect of CBD in this experimental setting. The mitochondrial collapse and cytochrome c release initiates a cascade of events leading to the activation of caspase-9 and later caspase-3 [35]. In our study, we did not find any attenuation of the H_2_O_2_-induced caspase-3 activity; however, we noticed a significant inhibitory effect of CBD on DNA fragmentation, as estimated by Hoechst 33342 staining. That suggests an involvement of caspase-3-independent mechanisms in neuroprotection mediated by CBD. Future studies should verify the engagement of AIF (apoptosis-inducing factor) in CBD-mediated neuroprotection since, after its translocation from mitochondria to the cytoplasm, it could evoke large-scale DNA fragmentation, leading to cell demise [36]. To this end, our previous data showed AIF translocation in the model of neuronal cell damage induced by H_2_O_2_ in SH-SY5Y cells [28], and similar findings were reported by Seong-Woon et al. [37] for primary cortical neurons. So far, no data has shown an association of CBD neuroprotection with the inhibition of AIF translocation. Since CBD acts on many targets, among which not only cannabinoid receptors could be included, but there are also other possible targets that could be involved in its neuroprotective effects (e.g., adenosine receptor subtype 2A, 5-HT_1A_, TVRP, PPARγ) [8]. Thus, it is not excluded that some of them could be responsible for the CBD-mediated neuroprotection observed against H_2_O_2_ neurotoxicity. However, this assumption needs to be verified in further experimental studies.

The ability of CBD in low micromolar concentrations to protect cells against oxidative stress-related damage was also confirmed in the SH-SY5Y cell model. Indeed, in our study, CBD in low concentration protected SH-SY5Y cells against H_2_O_2_ and 6-OHDA-evoked cell death. Protective effects of CBD against H_2_O_2_-evoked cell damage in SH-SY5Y cells were also observed by Yordanov et al. [24] but at higher concentrations (0.39 and 0.78 μM) than in our study (0.01 and 0.05 μM). It is not excluded that the medium composition during experiments could be responsible for these discrepancies in effective protective concentrations. However, in neuronally differentiated SH-SY5Y cells, no protective effects of CBD at a concentration of 2.5 μM against cell damage induced by H_2_O_2_, 6-OHDA, glycolaldehyde, or methylglyoxal were observed [32]. Thus, it may be hypothesized that neuroprotective mechanisms of CBD could engage the same prosurvival pathways as those induced by retinoic acid, e.g., PI3-K/Akt or ERK1/2 [38]. It should be noted that during the differentiation of SH-SY5Y cells, CBD (2.5 μM) sensitized the cells to detrimental effects of all redox-active drugs (H_2_O_2_, 6-OHDA, glycolaldehyde or methylglyoxal) [32]. Our data obtained in SH-SY5Y cells support reports showing the neuroprotective effects of CBD in other in vitro models of Parkinson’s disease. Thus, CBD protected the neural cells from apoptosis in vitro menstrual stromal cell-derived dopamine-like neurons (DALNs) exposed to paraquat, and this effect was accompanied by inhibition of caspase-3 activity [39]. CBD also reduces cell damage of undifferentiated and retinoic acid differentiated SH-SY5Y cells exposed to 1-methyl-4-phenylpyridinium (MPP+)—A neurotoxin which inhibits mitochondrial complex I, decreases ATP level and enhances ROS production [23,40]. In undifferentiated cells, protection was demonstrated for CBD at concentrations 25 and 50 μM and was associated with induction of autophagy and alleviation of mitochondrial dysfunction by upregulation of SIRT1 and inhibition of NF-κB and NOTCH pathways [23]. Whereas in differentiated cells, CBD was protective against MPP+-evoked cell damage at concentration 10 μM CBD via activation of ERK and AKT/mTOR pathways, reduction in Bax and the nuclear levels of PARP-1, inhibition of caspase-3 and inhibition of autophagy [40]. Moreover, the effects of CBD on ERK and autophagic pathways were attenuated by CB2 and TRPV1 but not by CB1 antagonists [40]. The above opposite effects of CBD in undifferentiated and retinoic acid-differentiated cells could raise a question of whether the differentiation process could affect cannabinoid pathways/receptors in SH-SY5Y cells. Up to now, it has been demonstrated that undifferentiated SH-SY5Y express the mRNA of enzymatic components of the endogenous cannabinoid system including diacylglycerol lipases (DAGLα and DAGLβ), monoacylglycerol lipase (MAGL), α/β-hydrolase domain containing 6 (ABHD6), ABHD12, N-acyl phosphatidylethanolamine-specific phospholipase D (NAPE-PLD), and fatty acid amide hydrolase (FAAH), but the CB1 and CB2 mRNA were expressed in relatively very low level [22]. Thus, cell lines expressing exogenous CB1 or CB2 were created, and they did not differ from parental cells with respect to proliferation, viability, or apoptosis, but they had an impact on neurite length [41]. Moreover, it has been shown that during the differentiation of SH-SY5Y cells, there is a decrease in the mRNA level of the endocannabinoid hydrolyzing enzyme MAGL [41]. Since we did not perform a mechanistic study, it is difficult to speculate if, and to what extent, CBD exerted its neuroprotective effects in SH-SY5Y cells via an endogenous cannabinoid system. This compound interacts with a high number of molecular targets, including G protein-coupled receptors (adenosine receptor subtype 2A, 5-HT_1A_, and GPR55), ligand-gated ion channels (TRPV), or PPARγ [8]. Thus, further mechanistic studies are needed to clarify the neuroprotective mechanisms of CBD observed in SH-SY5Y cells.

Our findings did not provide evidence for the direct effect of CBD on H_2_O_2_-induced increase in intracellular ROS level. However, some reports support the notion that the neuroprotective effects of CBD could result from its direct antioxidative properties [11]. Indeed, CBD was shown to be a potent antioxidant in the brain lipid oxidation assay [42]. These investigators also reported that antioxidant phenolic cannabinoids in concentrations 1 and 10 μM were also protective against oxidative stress in rat primary cerebellar granule cells and the HT22 and PC12 neuronal cell lines [42]. Furthermore, in our experimental settings, the concentration-dependent neurotoxic or neuroprotective CBD effects were clearly displayed. Surprisingly, in contrast to other reports, CBD in our study had no effect or only increased cortical neuron damage resulting from excitotoxic insults such as Glu and OGD. In fact, Glu neurotoxicity is accompanied by an increase in ROS production and could be ameliorated by antioxidants [43]. Therefore, it might be expected that the potent antioxidant–CBD should be protective, at least partly, in such models. The lack of protective effects of CBD in our in vitro model of ischemia (OGD) is in contrast to results obtained by Landucci et al. [44] and Lana et al. [45], who showed that CBD studied in single concentration 10 μM was neuroprotective in rat organotypic hippocampal slices exposed to OGD, acting, at least in part, via TRPV2 channels. This difference may be due to the fact that in our study, we used an isolated neuronal model containing only a small fraction of astrocytes (about 20%) and devoid of microglia cells, while Lana et al. [45] or Landucci et al. [44] used a model containing all cell phenotypes (neurons, astrocytes, and microglia). Indeed, CBD may affect neuronal survival indirectly through the modulation of astrocytes and microglia activity and morphology [9,46]. In this regard, it was found that CBD (10 μM) inhibited microglia activation and mitigated neuronal damage induced using KA in rat organotypic hippocampal slices [44]. CBD in high concentration (100 μM) showed neuroprotective effects also in microglia containing forebrain slices from newborn mice which underwent OGD-reducing glutamate and IL-6 (interleukin 6) concentration, and TNFalpha, COX-2 (cyclooxygenase 2), and iNOS (inducible nitric oxide synthase) expression, and these effects were mediated by CB2 and adenosine A2A receptors [47].

On the other hand, in a mouse hippocampal HT22 cell line, CBD prevented the OGD/reoxygenation-induced cell death, ameliorated intracellular ROS production and lipid peroxidation by enhancing mitochondrial bioenergetics and modulating glucose metabolism via pentose-phosphate pathway [48]. In human brain vascular pericytes exposed to OGD, both natural and synthetic CBD (100 nM) attenuated cellular damage to a similar extent as measured using lactate dehydrogenase at 24 h [49]. This study confirms the notion that submicromolar concentration of CBD is sufficient to exert neuroprotective effects. Another study showed that CBD-enriched non-psychotropic *Cannabis sativa* L. extract markedly protected SH-SY5Y cells from Glu-induced decrease cell viability and, via ERK modulation, counteracted the alterations in brain-derived neurotrophic factor levels [50]. However, the contribution of CBD to the protective effects of the whole cannabis phytocomplex was not established. The concentration-dependent opposite effects of CBD on neuronal survival suggest that its blood and brain concentration needs to be controlled. In connection with this, the CBD-loaded nanocarriers are currently being designed to overcome its poor oral bioavailability and improve the therapeutic efficacy of this compound [24,51,52]. Although clinical trials showed that CBD has an excellent safety profile and could be beneficial in the treatment of various neurological and neuropsychiatric conditions such as epilepsy, chronic pain, inflammation, anxiety, and neurodegenerative diseases, some undesired effects of this drug on hepatic drug metabolism and transport, fertility and in vitro cell viability were reported [53,54,55]. Our study provided evidence that CBD in submicromolar or low micromolar concentration is safe and neuroprotective, but in higher concentrations, it has detrimental effects on cell viability and may enhance the potency of some cell-damaging factors. We are fully aware that a translational value of the in vitro data obtained at a specific experimental setting may be questioned. Nevertheless, the data support the notion that further studies on possible side effects of CBD and monitoring CBD blood concentrations are justified, especially when taking into account the extremely complex neurochemical mechanism of CBD action, which involves at least 56 molecular targets, including metabotropic and ionotropic receptors, nuclear receptors, and enzymes [8,56].

## 5. Conclusions

Results of this study indicate that CBD has dual effects on oxidative stress-induced neuronal death-in low concentrations, it is neuroprotective, but in higher concentrations, it displays neurotoxic activity. On the other hand, in excitotoxic-related models, CBD was ineffective or enhanced cell damage. Our data support the notion that the neuroprotective effects of CBD strongly depend on its concentration and experimental model of neuronal damage.

## Data Availability

The raw data that support the findings of this study are available from the corresponding author upon reasonable request.

## Abbreviations

6-OHDA—6-hydroxydopamine; AMPA—α-amino-3-hydroxy-5-methyl-4-isoxazolepropionic acid; CBD—cannabidiol; FBS—fetal bovine serum; Glu—glutamate; H_2_O_2_—hydrogen peroxide; KA—kainic acid; LDH—lactate dehydrogenase; MTT—3-[4,5-dimethylthiazol-2-yl]-2,5-diphenyltetrazolium bromide; NMDA—N-methyl-D-aspartate; OGD—oxygen glucose deprivation; RNS—reactive nitrogen species; ROS—reactive oxygen species; WST-1—Water-Soluble Tetrazolium.
